# 682. Implantable Cardioverter-Defibrillator Lead Vegetation with Long-Standing *Actinomyces neuii* Bacteremia

**DOI:** 10.1093/ofid/ofab466.879

**Published:** 2021-12-04

**Authors:** Mark Irwin, Steven Tilem, Charlie Ervin, Fernando de la Serna, Rahul Sampath

**Affiliations:** 1 Carolinas Health Care System Blue Ridge, Morganton, North Carolina; 2 Carolinas Healthcare System, Morganton, North Carolina; 3 Carolinas HealthCare Systems BlueRidge, Morganton, NC

## Abstract

**Background:**

Endocarditis caused by *Actinomyces* species is uncommon with only 30 cases reported in contemporary literature.

**Methods:**

We present a novel case of cardiovascular implantable electronic device (CIED) endocarditis secondary to infection by *Actinomyces neuii* – a unique non-branching member of the species that grows in both anaerobic and aerobic media.

**Results:**

Our patient, a 51-year-old female, with a history of implantable cardioverter-defibrillator (ICD) placement 17 years prior for heart failure, presented with six weeks of fevers and rigors. She was referred to the infectious disease clinic for evaluation of pyrexia of unknown origin. Her examination was unremarkable, and the ICD pocket was uninflamed. Her initial labs revealed mildly elevated inflammatory markers and renal insufficiency. Blood cultures were positive for slow-growing non-branching gram-positive rods in both aerobic and anaerobic media. These were identified as *Actinomyces neuii* by mass spectrometry. Review of outside records showed positive blood cultures with *Actinomyces neuii* at another facility two weeks prior to our evaluation which were not acted upon and thought to be bacterial contamination. The patient was further evaluated with a transesophageal echocardiogram that demonstrated a 3.3 x 2.2cm mobile vegetation attached to the ICD lead. She subsequently underwent removal of her Saint Jude cardiac resynchronization therapy defibrillator and leads using laser and snaring techniques, but the tail end of the ventricular lead fractured and could not be retrieved. The ICD pocket was also found to be infected. A planned 6-week course of IV ampicillin was interrupted by 2 weeks of ceftriaxone for treatment of an intercurrent lower respiratory tract infection. The patient regained her baseline health and was discharged 2 weeks after ICD removal with a LifeVest. She is to complete 12 months of oral amoxicillin therapy after completion of IV antibiotics in view of retained lead fragment, and long standing *Actinomyces* bacteremia - consistent with published management strategies.

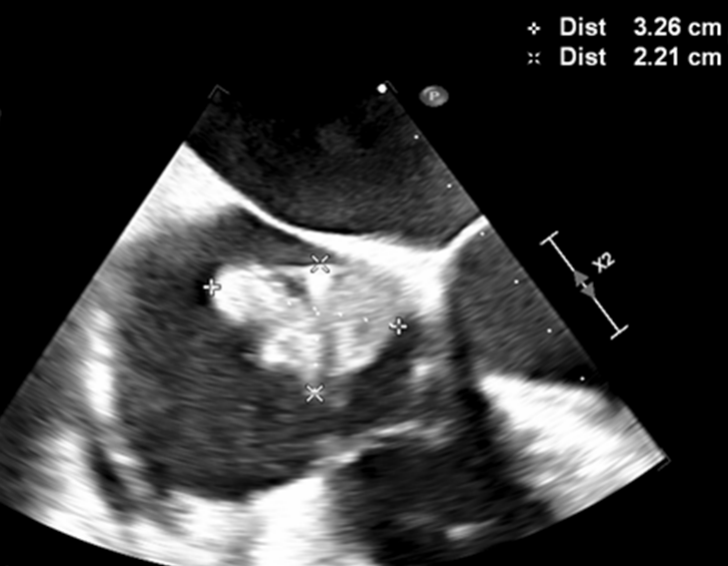

Figure 1. Transesophageal echocardiogram demonstrating size of vegetation.

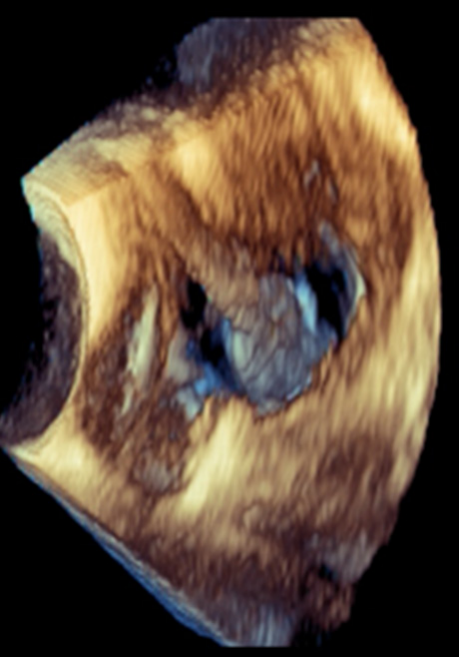

Figure 2. Three-dimensional view demonstrating vegetation on the ICD lead.

**Conclusion:**

Here we describe the first known case of *Actinomyces neuii* CIED endocarditis with a large lead vegetation and long-standing bacteremia, presenting as pyrexia of unknown origin.

**Disclosures:**

**All Authors**: No reported disclosures

